# Profile and Migration of Members of Residency Programs in Family Medicine

**DOI:** 10.11606/s1518-8787.2022056003450

**Published:** 2022-04-11

**Authors:** Ana Paula Tussi Leite, Scheila Mai, Alice Paul Waquil, Elvira Alicia Aparicio Cordero, Vitória Silva da Rosa, Carmen Vera Giacobbo Daudt, Brenda Wander, Maria Eugênia Bresolin Pinto, Isabel Brandão Correia, Thiago Dias Sarti

**Affiliations:** I Hospital Moinhos de Vento Programa de Apoio ao Desenvolvimento Institucional do Sistema Único de Saúde Porto Alegre RS Brasil Hospital Moinhos de Vento. Programa de Apoio ao Desenvolvimento Institucional do Sistema Único de Saúde. Porto Alegre, RS, Brasil; II Universidade do Vale do Rio dos Sinos Escola de Saúde São Leopoldo RS Brasil Universidade do Vale do Rio dos Sinos. Escola de Saúde. São Leopoldo, RS, Brasil; III Universidade Federal de Ciências da Saúde de Porto Alegre Departamento de Saúde Coletiva Porto Alegre RS Brasil Universidade Federal de Ciências da Saúde de Porto Alegre. Departamento de Saúde Coletiva. Porto Alegre, RS, Brasil; IV Universidade de Pernambuco Faculdade de Ciências Médicas Recife PE Brasil Universidade de Pernambuco. Faculdade de Ciências Médicas. Recife, PE, Brasil; V Universidade Federal do Espírito Santo Departamento de Medicina Social Vitória ES Brasil Universidade Federal do Espírito Santo, Departamento de Medicina Social. Vitória, ES, Brasil

**Keywords:** Physicians, Family, Internship and Residency, Family Practice, Primary Health Care

## Abstract

**OBJECTIVE:**

To describe the sociodemographic profile and analyze the migratory characteristics of the members of the Residency Programs in Family Medicine in 2020 in Brazil.

**METHODS:**

The study follows a cross-sectional observational design of a quantitative nature from the perspective of the members of the Residency Programs in Family Medicine. Questionnaires adapted for each participating group were developed, applied through an online platform.

**RESULTS:**

Most participants are female and white. Most supervisors and preceptors were residents of Residency Programs in Family Medicine, however, there are some who are not specialists in the field. Most participants are based in capitals or metropolitan regions. In relation to retention, 41.1% of supervisors and 73.1% of preceptors are affiliated to a program in the same municipality where they lived. For most resident physicians, the place of residence coincides with the place of birth and/or graduation (57.4%), and 48.5% are in the same place of graduation.

**CONCLUSIONS:**

The research reinforces the need for policies to promote the migration of residents to Residency Programs in Family Medicine outside capital cities and metropolitan regions, as well as encouraging the retention of graduates trained outside large urban centers so that they can contribute to distribution and provision of doctors where they are still needed.

## INTRODUCTION

In Brazil, primary health care (PHC) has the Family Health Strategy (ESF) as its main organizational model, which is the main gateway to the Unified Health System (SUS). The country followed the premise of the Alma-Ata Declaration (1978), which points to the strengthening of health systems through robust, resolute, and equitable PHC. Currently, there are approximately 43,369 teams in FHS, which are responsible for most (63.7%) of PHC coverage in the country^1,2^. On the other hand, the expansion of PHC as a strategy adopted to reorient SUS has not been accompanied by the provision of professionals, especially physicians, in sufficient quantity and quality. Only 7,149 (1.7%) of physicians are specialized in Family Medicine (FM)^3^.

FM is recognized for playing a central role. The physicians serving there are trained in the integral care of the person through a comprehensive practice, in addition to being able to support the primary care team in a series of health problems. Thus, FM promotes early intervention, cost-effectiveness and benefits to populations that would otherwise need to seek care far from their communities^4^.

Among the measures aimed at reducing gaps in the training of specialists to work in PHC, the expansion of residency vacancies in FM^5,6^ stands out. Partnerships between the Ministry of Education (MEC), the Ministry of Health (MS) and the health secretariats supported the consolidation and expansion of the Residency Programs in Family Medicine (PRMFC), investing in the combination of interests of the national manager in the implementation of federal policies and the role of municipal managers in composing a strengthened network for the formation of FM^7,8^.

In view of the recognized role of residency as an important field of provision of specialist physicians for the support and qualification of PHC, it is essential to identify the profile and characteristics of the migration of those involved with PRMFC, which may constitute an important diagnosis for human resource policies for SUS. In this context, the objective of this study is to describe the sociodemographic profile and analyze the migratory characteristics of PRMFC members in 2020 in Brazil.

## METHODS

This is an observational and cross-sectional study of a quantitative nature that presents an excerpt from the research entitled Characterization of Residency Programs in Family Medicine in Brazil (2020). Through the results of this research, we will approach aspects related to supervisors, preceptors, and residents.

Since a validated and standardized tool for research on PRMFC is unknown, questionnaires were developed, which had questions about sociodemographic and professional aspects, adapted for each participating group. Two pre-test phases of the questionnaire were conducted, with the contribution of FCM experts linked to the Brazilian Society of Family Medicine (SBMFC) and MS technicians.

According to data from the National Commission for Medical Residency (CNRM), in May 2019 there were 302 PRMFC with accreditation in Brazil. However, 53 of them reported that they were not active, which leads to a total of 249 programs. Therefore, through the CNRM list, a population of 249 supervisors and 1,329 residents is estimated. Following the criterion recommended in Brazilian legislation^9^ of one preceptor for every three residents, it is estimated that there are 443 preceptors.

The supervisors’ contacts were obtained through the PRMFC, SBMFC, residents and the MS list. The corresponding questionnaires were sent to supervisors and residents, with the Free Informed Consent Term, by e-mail and/or messaging application. For preceptors and residents, submission was made via supervisor. Data collection took place between January and April 2020, through the Research Electronic Data Capture (REDCap) online platform.

The categorical variables of each of the three participating groups were summarized through tables with absolute and relative frequencies, and the quantitative variables by mean and standard deviation. The chi-square test was used to compare proportions between groups. To assess the characteristics associated with the retention of supervisors and preceptors who attended FM residency in relation to the municipality where they are currently linked, a Poisson regression was used. The outcome used was fixation, and the characteristics included in the model were: group; gender; age; marital status; type of current municipality; and indicator of change between cities where they attended graduation and residency. As there was only one person with an undeclared gender, the observation was removed from the regression analysis; for the same reason, the observation whose marital status is widowed was grouped with the separate category. Thus, 221 observations of supervisors and preceptors who attended FM residency were included in the regression model. All analyzes were performed using R software, version 3.6.1, and a significance level of 5% was adopted.

The research was approved by the Research Ethics Committee of Hospital Moinhos de Vento, with report (CAAE: 22004819.8.0000.5330). All research participants signed the informed consent form. The data presented were obtained in partnership with the Ministry of Health, with the Support Program for Institutional Development of the Unified Health System (PROADI-SUS) as a source of funding.

## RESULTS

A total of 876 participants answered the questionnaires, of which 155 were supervisors (62.2%), 177 preceptors (40%) and 544 residents (40.9%).

All regions of the country are covered by PRMFC, of which the Southeast region has the highest concentration (n = 114; 45.8%) and municipalities with programs (n = 78; 49.7%), while the North regions (n = 17; 6.8%) and the Midwest have the lowest concentrations (n = 13; 5.2%). The Southeast was the region with the highest frequency of participants in all groups, followed by the Northeast region, for supervisors and residents, and the South, for preceptors (Table 1).

**Table 1:** Distribution of active and participating Family Medicine Residency Programs by region and group. Brazil, 2020.

Place of affiliation, n (%)	Active PRMFC	Municipalities with PRMFC	Responding supervisors	Responding preceptors	Responding residents
North	17 (6.8)	11 (7.0)	10 (6.5)	9 (5.1)	32 (5.9)
Northeast	53 (21.3)	30 (19.1)	39 (25.2)	30 (16.9)	105 (19.3)
Midwest	13 (5.2)	8 (5.1)	10 (6.5)	19 (10.7)	59 (10.8)
Southeast	114 (45.8)	78 (49.7)	64 (41.3)	69 (39.0)	245 (45.0)
South	52 (20.9)	30 (19.1)	32 (20.6)	50 (28.2)	103 (18.9)
**Total**	**249 (100)**	**157 (100)**	**155 (100)**	**177 (100)**	**544 (100)**

Source: Elaboration based on data from the research “Characterization of Residency Programs in Family Medicine in Brazil.”

PRMFC: Family Medicine Residency Programs.

The evaluation of the participants’ responses showed a predominance of female (n = 533; 60.9%) and white people (n = 597; 68.2%). The age distribution of the resident physicians participating showed that 94.7% were in the age group up to 39 years, with an average of 30.7 (standard deviation = 5.1) and single (n = 347; 63.8 %). Supervisors and preceptors were mostly married (n = 254; 76.5%) and were between 30 and 49 years old (n = 246; 74.3%), with the average age of supervisors being 44.7 (standard deviation = 10.1) and of preceptors 39 (standard deviation = 8.5) years old (Table 2).

**Table 2:** Distribution of sociodemographic characteristics of supervisors, preceptors, and resident physicians of the PRMFC. Brazil, 2020.

Feature	Supervisor (n = 155)	Preceptor (n = 177)	Resident (n = 544)
Age group, n (%)			
Up to 29 years	6 (3.9)	20 (11.4)	310 (57.2)
30–39	54 (34.8)	81 (46.0)	203 (37.5)
40–49	56 (36.1)	55 (31.3)	23 (4.2)
50 years or older	39 (25.2)	20 (11.4)	6 (1.1)
Total	155	176	542
Gender, n (%)			
Female	82 (52.9)	102 (57.6)	349 (64.3)
Male	73 (47.1)	74 (41.8)	192 (35.4)
Other	0 (0.0)	1 (0.6)	2 (0.4)
Total	155	177	543
Marital status, n (%)			
Single	24 (15.5)	35 (19.8)	347 (63.8)
Married	123 (79.4)	131 (74.0)	190 (34.9)
Divorced	6 (3.9)	10 (5.6)	7 (1.3)
Widower	2 (1.3)	1 (0.6)	0 (0.0)
Total	155	177	544
Race/Color, n (%)			
Black	5 (3.2)	2 (1.1)	27 (5.0)
Brown	33 (21.3)	48 (27.1)	138 (25.4)
White	114 (73.5)	125 (70.6)	358 (65.8)
Yellow	3 (1.9)	1 (0.6)	6 (1.1)
Indigenous	0 (0.0)	0 (0.0)	2 (0.4)
Not declared	0 (0.0)	1 (0.6)	13 (2.4)
Total	155	177	544

Source: Elaboration based on data from the research “Characterization of Residency Programs in Family Medicine in Brazil.”

PRMFC: Family Medicine Residency Programs.

Regarding education, most supervisors (n = 105; 68.2%) and preceptors (n = 114; 64.8%) had graduated from public higher education institutions (federal or state), representing 66.4% of the participants in these groups. Among residents, most (n = 321; 59.1%) attended private institutions (private, philanthropic or community).

When analyzing the specialists by medical residency and by residency and proof of SBMFC title, most supervisors (n = 92; 59.8%) and preceptors (n = 130; 73.5%) had the experience of attending the residency in FM, that is, 67.1% were residents of PRMFC. However, there are supervisors (n = 27; 17.5%) and preceptors (n = 18; 10.2%) who are not FM. Statistical analysis indicates that the proportions of supervisors and preceptors, in relation to obtaining the title, are significantly different (p-value = 0.023), with the main difference in the category of specialists by residence, which has proportions of 41.6 % of supervisors and 57.6% of preceptors (Table 3).

**Table 3:** Acquisition of the title of specialist in Family Medicine from the supervisor and preceptor. Brazil, 2020.

FCM specialist, n (%)		Supervisor	Preceptor	p
Yes	By residency	64 (41.6)	102 (57.6)	0.023
	By title	35 (22.7)	29 (16.4)	
	By residency and title	28 (18.2)	28 (15.8)	
Not FCM		27 (17.5)	18 (10.2)	
**Total**		**154 (100)**	**177 (100)**	-

Source: Elaboration based on data from the research “Characterization of Residency Programs in Family Medicine in Brazil.”

FM: Family Medicine.

Regarding the completion of residency and obtaining a title in another medical specialty recognized by the Federal Council of Medicine (CFM), it is observed that 34.4% (n = 53) of supervisors have another degree, the most frequent being in Pediatrics and Preventive and Social Medicine. Among preceptors (n = 32; 18.1%), the most cited were Acupuncture and Geriatrics. And among residents (n = 42; 7.7%) Pediatrics, Internal Medicine and Preventive and Social Medicine.

Of the 27 supervisors who are not FM, the other most frequent specialties were Preventive and Social Medicine (n = 6; 22.2%), Pediatrics (n = 4; 14.8%) and Internal Medicine (n = 3; 11, 1%), and 5.2% reported that they did not attend a residency or have a degree in another specialty. Among preceptors who are not FM, the only other specialty reported by more than one preceptor was Internal Medicine (n = 2; 11.1%), and 7.3% of the total preceptors do not have any specialty.

Regarding the time of exercise of participating supervisors and preceptors in their current role, more than a half have held the position for less than 3 years, and for both groups, about 25% have up to one year, and approximately 35% have between 2 and 3 years in the role.

Considering only the supervisors who attended the residency in FM, 25.6% are currently linked to a PRMFC in the same municipality where they were born. The same is true for 43.1% of preceptors who did residency in FM and for 33% of residents, and the analysis indicates that these proportions between the groups are significantly different. Regarding graduation, for the three groups, about 50% of the participants are linked to a PRMFC in the same municipality where they graduated. In terms of having a link with a PRMFC in a different municipality from the municipality where they were born, attended graduation and residency - in the case of supervisors and preceptors - supervisors (35.6%) and residents (42.6%) have the highest percentages and the difference between the proportions of the three groups is significant. In addition, 41.1% of supervisors and 73.1% of preceptors are currently linked to a PRMFC in the same municipality where they were residents, and the analysis indicates that the proportions are significantly different (Table 4).

**Table 4:** Migration of supervisors and preceptors who attended residency in FM and resident physicians. Brazil, 2020.

Municipality	Supervisor (n = 90)	Preceptor (n = 130)	Resident^a^ (n = 542)	p
Based in the same municipality of birth.	23 (25.6)	56 (43.1)	179 (33.0)	**0.020**
Based in the same municipality of graduation.	40 (44.4)	65 (50.0)	263 (48.5)	0.706
Based in the same municipality of FCM residency.	37 (41.1)	95 (73.1)	-	**< 0.001**
Based in a different municipality of birth, graduation, and residency.	32 (35.6)	23 (17.7)	231 (42.6)^a^	**< 0.001**

Source: Elaboration based on data from the research “Characterization of Residency Programs in Family Medicine in Brazil.”

^a^ For residents, a municipality is considered different from the municipalities of birth and graduation.

FM: Family Medicine.

Most residents are in metropolitan regions (n = 327; 60.1%), and, among them, just over half remain in the same city where they graduated (n = 173; 53.1%). Among the residents where the PRMFC is in the countryside, the majority switched cities between graduation and residency (n = 126; 58.3%).

When analyzing the type of municipality of the participants who attended FM residency, there is no significant difference between groups (p-value = 0.702), with 51.1% of supervisors (n = 47) and 54.6% of preceptors (n = 71) are linked to PRMFC in metropolitan regions. Among supervisors who are currently in metropolitan regions, half (n = 22; 48.9%) settled in the municipality of residence, while among supervisors who are currently in municipalities in the countryside, the fixation is lower (n = 15; 33.3%), and two supervisors did not inform the place where they attended residency. Among preceptors, who have a higher percentage of tenure than supervisors, the permanence in the same municipality of residence is also greater when the municipality is a metropolitan region (n = 56; 78.8%) than among preceptors in the countryside (n=39; 66.1%).

A Poisson regression was used to assess the association between the retention of supervisors and preceptors who attended FM residency in relation to the municipality they are currently affiliated to and the characteristics: group; gender; age; marital status; type of current municipality; indicator of city change where they attended graduation and residency. With the adjustment of the model, only the variables group and migration in relation to the city of graduation proved to be significant. Thus, it is possible to conclude that the retention of preceptors is 1.7 times that of supervisors (95%CI: 1.14–2.49), keeping the other characteristics constant. Likewise, the prevalence of settling for people who changed cities between graduation and residency is 1.5 times the prevalence among people who did not change cities, with a confidence interval between 1.07 and 2.22, keeping the other constant features. Among supervisors and preceptors who attended FM residency and settled in the municipality (41.1% and 73.1% respectively), most are in capital cities or metropolitan regions (59.5% and 58.9%, respectively) (Table 4).

On the other hand, with regard to supervisors who moved to another municipality (n = 53; 58.9%) between their residence in FM and their current location, 40.4% attended residency in the capital or metropolitan region and migrated to a municipality in the countryside; 7.7% did the opposite; 36.5% were in a capital or metropolitan region and moved to another municipality of the same type; and 15.4% of supervisors moved between municipalities in the countryside. The same happens for preceptors who changed cities (n = 35; 26.9%) between their residence in FM and their current location: 40% migrated to the countryside; 5.7% went from the countryside to the capital or metropolitan region; 37.1% moved between capitals or metropolitan region; and 17.1% moved between municipalities in the countryside.

A portion of the supervisors who attended the FM residency (n = 22; 24.4%) are working in the same PRMFC where they were a resident, which represents 59.5% of those who stayed in the same municipality where they attended residency. Among the preceptors, the majority (n = 78; 60%) are working in the same PRMFC where they attended residency, representing 82.1% of those who remained in the municipality.

Most supervisors who are not FM are in the countryside (n = 21; 77.8%), with only 38.1% of these being in the same municipality where they graduated. Among those who are in the capital or metropolitan region, 50% remain in the city of graduation. The same occurs among supervisors who are FM only by title: most are in the countryside (n = 22; 62.9%) and 13.6% of them are in the same municipality where they attended graduation. Among those who are in the capital, 46.2% remain in the city of graduation. Among both non-FM and title-only preceptors, about 55% are in metropolitan areas. Among those who are not FM, approximately 40% remained in the city of graduation. Among specialists by title, 50% of those currently in capitals or metropolitan regions remained in the same municipality, and only 15.4% of preceptors who are in the countryside settled in the municipality of graduation.

## DISCUSSION

Characterizing the profile and migration of medical residency members can support representative entities in the formulation and reorganization of adequate policies that are compatible with reality, especially regarding the training of specialized human resources. This refers especially to the field of FM, considering that in Brazil there are few professionals with such training^10^.

Most participants (60.9%) are female, portraying the female presence in the FM specialty, which is also observed in countries such as Canada, Spain and the United Kingdom^11^, as well as in other areas of medicine, demonstrating the feminization of the medical career^3,10,14^. The average age of physicians in Brazil is 44.6 years, being 41.7 among the FM^3^, which parallels the data from the USA^15^, where the age group of the FM is in agreement with the averages found in this research among supervisors and preceptors.

The research demonstrates changes in relation to the institutions that train physicians, with most supervisors and preceptors having graduated from a federal or state public institution. Most residents (59.1%) attended a private philanthropic or community institution. These data corroborate the study that evaluated the distribution and expansion of medical courses in Brazil, noting that most schools (58.5%) and undergraduate places (65.8%) were under private management^16^.

In view of the training of specialist physicians, the study observed that 17.5% of supervisory participants lack a title in FM. Comparative data are unknown in the literature, but this percentage is believed to be high, considering the Brazilian legislation that describes that the supervisor and preceptors of the PRMFC should preferably be specialists with a certificate of residency in FM and/or certification of the accredited specialty or issued by SBMFC.

We can see that 10.2% of the responding preceptors lack the FM specialty, and 7.3% lack any specialty, which is worrying, given the quality of resident training^17^. The European Academy of Teachers in General Practice (EURACT), formed by preceptors in FM, with a long trajectory in in-service teaching^18^ and concerned with the quality of training, recommends that the preceptor has proven training in the specialty and completed teacher training, among other criteria^19^. For the above, specialization and teacher training are fundamental points, which should be encouraged and even required in the selection of preceptors of the PRMFC.

According to the Medical Demography in Brazil 2020, the country has a doctor rate (2.38 doctors per 1,000 inhabitants) close to developed countries, such as the USA (2.6), Canada (2.8) and the United Kingdom (3)^3,11^. Despite this number, Brazil still has great inequality in the distribution of the medical population between different geographic levels and between different medical specialties^3,20,21^. As for the distribution according to specialty, there are not enough doctors in specialties considered strategic for SUS, as FM, which in 2020 had only 7,149 doctors, that is, 1.7% of the country’s total specialists, a ratio of 3.4 per 100,000 inhabitants^3,22^, contrary to what is found in other countries, such as Spain, where 31% of physicians are working in PHC, a ratio of 87.84 physicians per 100,000 inhabitants^12^.

Reinforcing inequalities in the distribution of doctors throughout the national territory, there are capitals with more than 13 doctors per 1,000 inhabitants and regions in the interior of the Northeast with values below 1 doctor per 1,000 inhabitants^3^. These data are reaffirmed in the survey, in which the professional connection of the participants reveals that more than half of the preceptors and residents are affiliated to capitals. This is a global trend in the migration of health professionals in which the settlement has been concentrated in large urban centers and economic centers, with better working conditions, better infrastructure, and equipment, in addition to being places of concentration of teaching and health establishments^10,21,23^.

For preceptors, there is a high rate of fixation in the same municipality where they performed the PRMFC (73.1%). This result, as evidenced in the literature, shows that the place of residence favors the establishment of medical professionals, who tend to remain in the place where they performed their residence^10,21,24,25^. However, when analyzing the group of supervisors, most migrated from the municipality after completing their residency in FM, with 40.4% migrating from capitals/metropolitan region to the countryside, which may indicate that the expansion of PRMFC in places beyond the capital can contribute to the establishment of supervising physicians, as the presence of a supervisor is necessary for the program to function.

For most resident physicians, the place of residence coincides with the place of birth and/or graduation (57.4%), and 48.5% are in the same place of graduation. Silva et al.^26^ also identified this trend, revealing that 37% of the students want to settle in the same place as their graduation, 28% want to return to the city of birth and 20% want to live in a smaller urban center. These findings reinforce the importance of considering the geographic distribution of medical schools and residency programs^21,27^.

As for the time of work, more than half of the supervisors and preceptors have been working in the role for less than 3 years. We can infer that there is a moderate turnover in these functions, which may reflect a lack of appreciation and support for these professionals. In the case of preceptors, since 2016 there have been training policies that encourage the preceptor’s specialization, especially in FM, aiming to guarantee the supply of doctors with specific training in preceptorship, sufficient for the expansion, with quality, of the residency in FM^9^. It is believed that this type of training can establish the professional in the preceptor role.

It is concluded, then, that the research achieved its objective of characterizing the sociodemographic profile and analyzing the migratory characteristics of the members of the PRMFC in 2020 in Brazil, pointing out important themes for the strengthening of PHC, such as the provision and retention of specialists in FM, who, for the most part, are female, young adults and, more recently, graduated from private education institutions, who have sought additional training beyond the FM residency and perform more than one work activity.

As for the setting of participants, resident physicians are linked to large urban centers, many in the same place of birth and/or graduation. For the preceptors, the settlement occurs in the same municipality where the FM residency was attended. However, when it occurs, the process of migration from the municipality in which the residency took place is greater for municipalities in the interior than for the capital and metropolitan region.

One of the limitations of this study is the fact that the proportion of respondents from some regions was, in relative terms, higher compared to other regions. It is also important to emphasize that this is a cross-sectional study, which portrays the analysis of the participants in a changing context static.

Even so, the data presented here reinforce the need for permanent dialogue and strategies that involve distinct levels of management, in an attempt to enable and expand human resources policies in FM and the qualification of professionals involved with residency programs, such as preceptors and supervisors. Finally, we recommend policies that prioritize measures to promote the migration of residents to PRMFC who are distributed in locations outside the capitals and metropolitan regions, as well as encourage the establishment in these locations, so that they can contribute to the distribution and provision of doctors where it is still necessary.

## References

[1] Ministério da Saúde (BR). Portaria Nº 2.436 de 21 de setembro de 2017. Aprova a Política Nacional de Atenção Básica, estabelecendo a revisão de diretrizes para a organização da Atenção Básica, no âmbito do Sistema Único de Saúde. Diário Oficial da União. 22 set 2017; Seção 1:1.

[2] Ministério da Saúde (BR). e-Gestor Atenção Básica - Informação e Gestão da Atenção Básica. e Gestor AB. Cobertura da Atenção Básica. Brasília, DF; c2121 [cited 2020 Oct 27]. Available from: https://egestorab.saude.gov.br/paginas/acessoPublico/relatorios/relHistoricoCoberturaAB.xhtml

[3] Scheffer M, coordenador. Demografia Médica no Brasil 2020. São Paulo: Departamento de Medicina Preventiva da Faculdade de Medicina da USP; Conselho Regional de Medicina do Estado de São Paulo; Conselho Federal de Medicina; 2020 [cited 2020 Oct 27]. Available from: https://www.fm.usp.br/fmusp/conteudo/DemografiaMedica2020_9DEZ.pdf.

[4] World Health Organization, United Nations Children’s Fund. A vision for primary health care in the 21st century: towards universal health coverage and the Sustainable Development Goals. Geneva (CH): WHO; UNICEF; 2018 [cited 2020 Oct 27]. (Technical Series on Primary Health Care). Available from: https://apps.who.int/iris/bitstream/handle/10665/328065/WHO-HIS-SDS-2018.15-eng.pdf?sequence=1&isAllowed=y

[5] Coelho Neto GC, Antunes VH, Oliveira A. A prática da Medicina de Família e Comunidade no Brasil: contexto e perspectivas. Cad Saude Publica. 2019;35(1):e00170917. 10.1590/0102-311X00170917 30652813

[6] Storti MMT, Oliveira FP, Xavier AL. A expansão de vagas de residência de Medicina de Família e Comunidade por municípios e o Programa Mais Médicos. Interface (Botucatu). 2017;21 Supl 1:1301-13. 10.1590/1807-57622016.0511

[7] Barrêto DS, Melo Neto AJ, Figueiredo AM, Sampaio J, Gomes LB, Soares RS. The More Doctors Program and Family and Community Medicine residencies: articulated strategies of expansion and interiorization of medical education. Interface (Botucatu). 2019;23 Supl 1:e180032. 10.1590/Interface.180032

[8] Zarpelon LFB, Terencio ML, Batista NA. Integração ensino-serviço no contexto das escolas médicas brasileiras: revisão integrativa. Cienc Saude Coletiva. 2018;23(12):4241-8. 10.1590/1413-812320182312.32132016 30540007

[9] Ministério da Saúde (BR); Ministério da Educação. Portaria Interministerial Nº 1. 618, de 30 de setembro de 2015. Institui, no âmbito do Sistema Único de Saúde (SUS), como um dos eixos do Programa Mais Médicos - Residência, o Plano Nacional de Formação de Preceptores para os Programas de Residência na modalidade Medicina Geral de Família e Comunidade, com o fim de subsidiar e assegurar instrumentos para o processo de expansão de vagas de residência em Medicina Geral de Família e Comunidade, nos termos da Lei nº 12.871, de 22 de outubro de 2013. Brasília, DF: MS; MEC; 2015 [cited 2020 Oct 27]. Available from: https://www.poderesaude.com.br/novosite/images/publicacoes_01.10.2015-I.pdf

[10] 0. Rodrigues ET, Forster AC, Santos LL, Ferreira JBB, Falk JW, Dal Fabbro AL. Perfil e trajetória profissional dos egressos da residência em Medicina de Família e Comunidade do Estado de São Paulo. Rev Bras Educ Med. 2017;41(4):604-14. 10.1590/1981-52712015v41n4RB20160084

[11] Organisation for Economic Co-operation and Development. Doctors. Paris (FR): OECD; c2021 [cited 2020 Oct 29]. Available from: https://data.oecd.org/healthres/doctors.htm

[12] Barber Pérez P, Lópes-Valcárcel BG. Estimación de la oferta y demanda de médicos especialistas. España 2018-2030. Palmas (ES): Universidad de Palmas de Gran Canaria; 2018 rev enero 2019 [cited 2020 Oct 27]. Available from: https://www.mscbs.gob.es/profesionales/formacion/necesidadEspecialistas/doc/20182030EstimacionOfertaDemandaMedicosEspecialistasV2.pdf

[13] Jefferson L, Bloor K, Maynard A. Women in medicine: historical perspectives and recent trends. Br Med Bull. 2015;114(1):5-15. 10.1093/bmb/ldv007 25755293

[14] Scheffer MC, Cassenote AJF. A feminização da medicina no Brasil. Rev Bioet. 2013 [cited 2020 Oct 27];21(2):268-77. Available from: https://www.scielo.br/j/bioet/a/XtCnKjggnr6gFR3bTRckCxs/?format=pdf&lang=pt

[15] Association of American Medical Colleges. Active physicians by sex and specialty, 2017. Washington, DC: AAMC; 2017 [cited 2020 Oct 27]. Available from: https://www.aamc.org/data-reports/workforce/interactive-data/active-physicians-sex-and-specialty-2017

[16] Oliveira BLCA, Lima SF, Pereira MUL, Pereira Júnior GA, Oliveira BLCA, Lima SF, et al. Evolução, distribuição e expansão dos cursos de medicina no Brasil (1808-2018). Trab Educ Saude. 2019;17(1):e0018317. 10.1590/1981-7746-sol00183

[17] Ministério da Educação (BR), Secretaria de Ensino Superior, Comissão Nacional de Residência Médica. Resolução CNRM nº 1, de 25 de maio de 2015. Regulamenta os requisitos mínimos dos programas de residência médica em Medicina Geral de Família e Comunidade - R1 e R2 e dá outras providências. Diário Oficial da União. 26 maio 2015; Seção 1:11.

[18] Walsh AE, Antao V, Bethune, C, Cameron, S, Cavett, T, Clavet, D, et al. Fundamental teaching activities in Family Medicine: a framework for faculty development. Mississauga (CA): College of Family Physicians of Canada; 2015 [cited 2020 Oct 29]. Available from: https://www.cfpc.ca/CFPC/media/Resources/Education/FTA_GUIDE_TM_ENG_Apr15_REV.pdf

[19] EURACT Specialist Training Committee. Selection of General Practice / Family Medicine (GP/FM) trainers/ractices and implementation of specialist training in GP/FM. Jerusalem (ISR); 2012.

[20] 0. Oliveira APC, Gabriel M, Dal Poz MR, Dussault G. Desafios para assegurar a disponibilidade e acessibilidade à assistência médica no Sistema Único de Saúde. Cienc Saude Coletiva. 2017;22(4):1165-80. 10.1590/1413-81232017224.31382016 28444043

[21] Berger CB, Dallegrave D, Castro Filho ED, Pekelman R. A formação na modalidade Residência Médica: contribuições para a qualificação e provimento médico no Brasil. Rev Bras Med Fam Comun. 2017;12(39):1-10. 10.5712/rbmfc12(39)1399

[22] Scheffer MC, Pastor-Valero M, Cassenote AJF, Compañ Rosique AF. How many and which physicians? A comparative study of the evolution of the supply of physicians and specialist training in Brazil and Spain. Hum Resour Health. 2020;18:30. 10.1186/s12960-020-00472-0 PMC7171868 32316989

[23] Veiga-Branco A, Ribeiro MI, Andrade AJ, Cadinha LCD, Pires RB, Façanha ALC, et al. Relationship between professional motivations and the expectation of staying at the same workplace: a cross-sectional descriptive study with physicians in Portugal. In: The 32nd International Business Information Management Association Conference; 2018; Sevilla, España. p. 670-86.

[24] Ney MS, Rodrigues PHA. Fatores críticos para a fixação do médico na Estratégia Saúde da Família. Physis. 2012;22(4):1293-311. 10.1590/S0103-73312012000400003

[25] Anisimowicz Y, Miedema B, Easley J, Bowes AE. Factors influencing Family Medicine resident retention and newly graduated physicians’ first practice location. J New Brunswick Stud. 2017 [cited 2020 Nov 3];8. Available from: https://journals.lib.unb.ca/index.php/JNBS/article/view/25884

[26] Silva MLAM, Amaral E, Machado HC, Passeri SMRR, Bragança JF. Influência de políticas de ação afirmativa no perfil sociodemográfico de estudantes de medicina de universidade brasileira. Rev Bras Educ Med. 2018;42(3):36-48. 10.1590/1981-52712015v42n3RB20170090r2

[27] Nunes MPT, Michel JLM, Brenelli SL, Haddad AE, Mafra D, Ribeiro ECO, et al. Distribuição de vagas de Residência Médica e de médicos nas regiões do país. Cad ABEM. 2011 [cited 2020 Nov 3];7:28-34. Available from: https://website.abem-educmed.org.br/wp-content/uploads/2019/09/CadernosABEM__Vol07.pdf

