# Supplementary material for: Profile and Migration of Members of Residency Programs in Family Medicine
Source: Rev Saude Publica. 2022 Apr 11;56:21. doi: 10.11606/s1518-8787.2022056003450 (PMC9004701; doi:10.11606/s1518-8787.2022056003450)
Supplement: Translation

# Translation: Perfil e migração de membros dos Programas de Residência em Medicina de Família e Comunidade

**Authors:** Ana Paula Tussi Leite, Scheila Mai, Alice Paul Waquil, Elvira Alicia Aparicio Cordero, Vitória Silva da Rosa, Carmen Vera Giacobbo Daudt, Brenda Wander, Maria Eugênia Bresolin Pinto, Isabel Brandão Correia, Thiago Dias Sarti

**Affiliations:** I Brasil Hospital Moinhos de Vento. Programa de Apoio ao Desenvolvimento Institucional do Sistema Único de Saúde. Porto Alegre, RS, Brasil; II Brasil Universidade do Vale do Rio dos Sinos. Escola de Saúde. São Leopoldo, RS, Brasil; III Brasil Universidade Federal de Ciências da Saúde de Porto Alegre. Departamento de Saúde Coletiva. Porto Alegre, RS, Brasil; IV Brasil Universidade de Pernambuco. Faculdade de Ciências Médicas. Recife, PE, Brasil; V Brasil Universidade Federal do Espírito Santo, Departamento de Medicina Social. Vitória, ES, Brasil

**Keywords:** Médicos de Família, Internato e Residência, Medicina de Família e Comunidade, Atenção Primária à Saúde

## Abstract

**OBJETIVO:**

Caracterizar o perfil sociodemográfico e analisar as características migratórias dos integrantes dos Programas de Residência em Medicina de Família e Comunidade em 2020 no Brasil.

**MÉTODOS:**

O estudo segue um delineamento observacional transversal de natureza quantitativa a partir da perspectiva dos integrantes dos Programas de Residência em Medicina de Família e Comunidade. Foram desenvolvidos questionários adaptados para cada grupo participante, aplicados por meio de plataforma on-line.

**RESULTADOS:**

A maioria dos participantes é do sexo feminino e de cor branca. A maioria dos supervisores e preceptores foi residente de Programas de Residência em Medicina de Família e Comunidade, contudo, há alguns que não são especialistas na área. A maior parte dos participantes está vinculada às capitais ou regiões metropolitanas. Em relação à fixação, 41,1% dos supervisores e 73,1% dos preceptores estão vinculados a um programa no mesmo município onde foram residentes. Para a maioria dos médicos residentes, o local da residência coincide com o local de nascimento e/ou graduação (57,4%), sendo que 48,5% estão no mesmo local de graduação.

**CONCLUSÕES:**

A pesquisa reforça a necessidade de políticas de promoção da migração de residentes para Programas de Residência em Medicina de Família e Comunidade fora das capitais e regiões metropolitanas, bem como estimula a fixação dos egressos formados fora dos grandes centros urbanos para que eles possam contribuir com a distribuição e com o provimento de médicos onde ainda é necessário.

## INTRODUÇÃO

No Brasil, a atenção primária à saúde (APS) tem como principal modelo de organização a Estratégia Saúde da Família (ESF), que se constitui como a principal porta de entrada do Sistema Único de Saúde (SUS). O país seguiu a premissa da Declaração de Alma-Ata (1978), que aponta para o fortalecimento dos sistemas de saúde por meio de uma APS robusta, resolutiva e equânime. Atualmente, há aproximadamente 43.369 equipes na ESF, que são responsáveis pela maior parte (63,7%) da cobertura de APS no país^1,2^. Em contrapartida, a expansão da APS enquanto estratégia adotada para reorientação do SUS não vem sendo acompanhada de provimento de profissionais, sobretudo médicos, em quantidade e qualidade suficientes. Apenas 7.149 (1,7%) dos médicos são especializados em Medicina de Família e Comunidade (MFC)^3^.

A MFC é reconhecida por desempenhar um papel central. Os médicos que atendem nela são treinados para o cuidado integral da pessoa por meio de uma prática abrangente, além de serem capazes de apoiar a equipe de cuidados primários em uma série de problemas de saúde. Assim, a MFC promove intervenção precoce, promoção custo-efetividade e benefícios às populações que, de outra forma, precisariam buscar atendimento longe de suas comunidades^4^.

Entre as medidas que visam a redução das lacunas na formação de especialistas para atuar na APS, destaca-se a ampliação das vagas de residência em MFC^5,6^. As parcerias entre o Ministério da Educação (MEC), o Ministério da Saúde (MS) e as secretarias de saúde deram suporte à consolidação e à ampliação dos Programas de Residência em Medicina de Família e Comunidade (PRMFC), investindo na combinação de interesses do gestor nacional na implementação de políticas federais e do protagonismo dos gestores municipais em compor uma rede fortalecida para formação de MFC^7,8^.

Tendo em vista o reconhecido papel da residência como um importante campo de provimento de médicos especialistas para a sustentação e qualificação da APS, torna-se imprescindível a identificação do perfil e das características da migração dos envolvidos com os PRMFC, que poderá constituir um importante diagnóstico para políticas de recursos humanos para o SUS. Nesse contexto, o objetivo deste estudo é caracterizar o perfil sociodemográfico e analisar as características migratórias dos integrantes dos PRMFC em 2020 no Brasil.

## MÉTODOS

Este é um estudo observacional e transversal de natureza quantitativa que apresenta um recorte da pesquisa intitulada Caracterização dos Programas de Residência em Medicina de Família e Comunidade no Brasil (2020). Por meio dos resultados desta pesquisa abordaremos os aspectos relacionados aos supervisores, preceptores e residentes.

Uma vez que se desconhece uma ferramenta validada e padronizada para pesquisa de PRMFC, foram desenvolvidos questionários, que contavam com questões sobre aspectos sociodemográficos e profissionais, adaptados para cada grupo participante. Foram realizadas duas fases de pré-teste do questionário, que contaram com a contribuição de experts em MFC vinculados à Sociedade Brasileira de Medicina de Família e Comunidade (SBMFC) e técnicos do MS.

Segundo dados da Comissão Nacional de Residência Médica (CNRM), em maio de 2019 havia 302 PRMFC com credenciamento no Brasil. Contudo, 53 deles informaram que não estavam ativos, o que leva a um total de 249 programas. Portanto, por meio da lista da CNRM, estima-se uma população de 249 supervisores e 1.329 residentes. Seguindo o critério preconizado na legislação brasileira^9^ de um preceptor para cada três residentes, estima-se a existência de 443 preceptores.

Os contatos dos supervisores foram obtidos por meio dos PRMFC, da SBMFC, dos residentes e pela lista do MS. O envio dos questionários correspondentes aos supervisores e residentes, com o termo de consentimento livre e esclarecido, foi feito por e-mail e/ou aplicativo de mensagem. Para preceptores e residentes, o envio foi feito via supervisor. A coleta de dados ocorreu entre janeiro e abril de 2020, por meio da plataforma on-line Research Electronic Data Capture (REDCap).

As variáveis categóricas de cada um dos três grupos participantes foram sumarizadas por meio de tabelas com frequências absolutas e relativas, e as variáveis quantitativas por média e desvio padrão. O teste Qui-quadrado foi utilizado para comparar proporções entre os grupos. Para avaliar as características associadas à fixação de supervisores e preceptores que cursaram a residência em MFC em relação ao município onde estão atualmente vinculados foi utilizada uma regressão de Poisson. O desfecho utilizado foi a fixação, e as características incluídas no modelo foram: grupo; gênero; idade; estado civil; tipo de município onde está atualmente; e indicador de mudança entre municípios onde cursou a graduação e a residência. Por haver apenas uma pessoa com gênero não declarado, a observação foi retirada da análise de regressão; pelo mesmo motivo, a observação cujo estado civil é viúvo foi agrupada com a categoria separado. Assim, foram incluídas 221 observações de supervisores e preceptores que cursaram residência em MFC no modelo de regressão. Todas as análises foram realizadas no software R, versão 3.6.1, e foi adotado um nível de significância de 5%.

A pesquisa foi aprovada pelo Comitê de Ética em Pesquisa do Hospital Moinhos de Vento, com parecer (CAAE: 22004819.8.0000.5330). Todos os participantes da pesquisa assinaram o termo de consentimento livre e esclarecido. Os dados apresentados foram obtidos em parceria com o MS, tendo o Programa de Apoio ao Desenvolvimento Institucional do Sistema Único de Saúde (PROADI-SUS) como fonte de financiamento.

## RESULTADOS

Um total de 876 participantes responderam aos questionários, sendo 155 supervisores (62,2%), 177 preceptores (40%) e 544 residentes (40,9%).

Todas as regiões do país estão contempladas com PRMFC, sendo a região Sudeste a de maior concentração (n = 114; 45,8%) e de municípios com programas (n = 78; 49,7%), enquanto as regiões Norte (n = 17; 6,8%) e Centro-Oeste têm as menores concentrações (n = 13; 5,2%). O Sudeste foi a região com maior frequência de participantes em todos os grupos, seguido da região Nordeste, para supervisores e residentes, e Sul, para os preceptores (Tabela 1).

**Tabela 1:** Distribuição dos Programas de Residência de Medicina de Família e Comunidade ativos e participantes por regiões e grupo. Brasil, 2020.

Local de vínculo, n (%)	PRMFC ativos	Municípios com PRMFC	Supervisores respondentes	Preceptores respondentes	Residentes respondentes
Norte	17 (6,8)	11 (7,0)	10 (6,5)	9 (5,1)	32 (5,9)
Nordeste	53 (21,3)	30 (19,1)	39 (25,2)	30 (16,9)	105 (19,3)
Centro-Oeste	13 (5,2)	8 (5,1)	10 (6,5)	19 (10,7)	59 (10,8)
Sudeste	114 (45,8)	78 (49,7)	64 (41,3)	69 (39,0)	245 (45,0)
Sul	52 (20,9)	30 (19,1)	32 (20,6)	50 (28,2)	103 (18,9)
Total	249 (100)	157 (100)	155 (100)	177 (100)	544 (100)

Fonte: Elaboração a partir dos dados da pesquisa “Caracterização dos Programas de Residência em Medicina de Família e Comunidade no Brasil”.

PRMFC: Programas de Residência de Medicina de Família e Comunidade.

A avaliação das respostas dos participantes mostrou predomínio do sexo feminino (n = 533; 60,9%) e da cor branca (n = 597; 68,2%). A distribuição etária dos participantes médicos residentes mostrou que 94,7% estavam na faixa etária até 39 anos, com médias de 30,7 (desvio padrão = 5,1) e, em sua maioria, solteiros (n = 347; 63,8%). Supervisores e preceptores eram, em sua maioria, casados (n = 254; 76,5%) e tinham entre 30 e 49 anos (n = 246; 74,3%), sendo a média de idade dos supervisores 44,7 anos (desvio padrão = 10,1) e dos preceptores 39 (desvio padrão = 8,5) (Tabela 2).

**Tabela 2:** Distribuição das características sociodemográficas dos supervisores, preceptores e médicos residentes dos PRMFC. Brasil, 2020.

Característica	Supervisor (n = 155)	Preceptor (n = 177)	Residente (n = 544)
Faixa etária, n (%)			
Até 29 anos	6 (3,9)	20 (11,4)	310 (57,2)
30–39	54 (34,8)	81 (46,0)	203 (37,5)
40–49	56 (36,1)	55 (31,3)	23 (4,2)
50 anos ou mais	39 (25,2)	20 (11,4)	6 (1,1)
Total	155	176	542
Gênero, n (%)			
Feminino	82 (52,9)	102 (57,6)	349 (64,3)
Masculino	73 (47,1)	74 (41,8)	192 (35,4)
Outro	0 (0,0)	1 (0,6)	2 (0,4)
Total	155	177	543
Estado civil, n (%)			
Solteiro	24 (15,5)	35 (19,8)	347 (63,8)
Casado	123 (79,4)	131 (74,0)	190 (34,9)
Separado	6 (3,9)	10 (5,6)	7 (1,3)
Viúvo	2 (1,3)	1 (0,6)	0 (0,0)
Total	155	177	544
Raça/Cor, n (%)			
Preta	5 (3,2)	2 (1,1)	27 (5,0)
Parda	33 (21,3)	48 (27,1)	138 (25,4)
Branca	114 (73,5)	125 (70,6)	358 (65,8)
Amarela	3 (1,9)	1 (0,6)	6 (1,1)
Indígena	0 (0,0)	0 (0,0)	2 (0,4)
Não declarada	0 (0,0)	1 (0,6)	13 (2,4)
Total	155	177	544

Fonte: Elaboração a partir dos dados da pesquisa “Caracterização dos Programas de Residência em Medicina de Família e Comunidade no Brasil”.

PRMFC: Programas de Residência de Medicina de Família e Comunidade.

Quanto à escolaridade, a maioria dos supervisores (n = 105; 68,2%) e preceptores (n = 114; 64,8%) cursou a graduação em instituições de ensino superior públicas (federal ou estadual), representando 66,4% dos participantes desses grupos. Entre os residentes, a maior parte (n = 321; 59,1%) cursou em instituições privadas (particulares, filantrópicas ou comunitárias).

Quando analisados os especialistas por residência médica e por residência e prova de título da SBMFC, percebe-se que a maior parte dos supervisores (n = 92; 59,8%) e preceptores (n = 130; 73,5%) teve a experiência de cursar a residência em MFC, ou seja, 67,1% foram residentes do PRMFC. Entretanto, há supervisores (n = 27; 17,5%) e preceptores (n = 18; 10,2%) que não são MFC. A análise estatística indica que as proporções de supervisores e preceptores, em relação à obtenção do título, são significativamente diferentes (p-valor = 0,023), sendo que a principal diferença está na categoria de especialistas por residência, que tem proporções de 41,6% dos supervisores e 57,6% dos preceptores (Tabela 3).

**Tabela 3:** Obtenção do título de especialista em Medicina de Família e Comunidade do supervisor e preceptor. Brasil, 2020.

Especialista em MFC, n (%)		Supervisor	Preceptor	p-valor
Sim	Por residência	64 (41,6)	102 (57,6)	0,023
	Por título	35 (22,7)	29 (16,4)	
	Por residência e título	28 (18,2)	28 (15,8)	
Não é MFC		27 (17,5)	18 (10,2)	
Total		154 (100)	177 (100)	-

Fonte: Elaboração a partir dos dados da pesquisa “Caracterização dos Programas de Residência em Medicina de Família e Comunidade no Brasil”.

MFC: Medicina de Família e Comunidade.

Em relação à conclusão de residência e obtenção de título em outra especialidade médica reconhecida pelo Conselho Federal de Medicina (CFM), observa-se que 34,4% (n = 53) dos supervisores têm outra titulação, sendo as mais frequentes em Pediatria e Medicina Preventiva e Social. Entre os preceptores (n = 32; 18,1%), as mais citadas foram Acupuntura e Geriatria. E entre os residentes (n = 42; 7,7%) Pediatria, Clínica Médica e Medicina Preventiva e Social.

Dos 27 supervisores que não são MFC, as outras especialidades mais frequentes foram Medicina Preventiva e Social (n = 6; 22,2%), Pediatria (n = 4; 14,8%) e Clínica Médica (n = 3; 11,1%), e 5,2% informaram que não cursaram residência ou possuem título em outra especialidade. Entre os preceptores que não são MFC, a única outra especialidade informada por mais de um preceptor foi Clínica Médica (n = 2; 11,1%), e 7,3% do total de preceptores não possui qualquer especialidade.

Em relação ao tempo de exercício dos participantes supervisores e preceptores na respectiva função atual, mais da metade ocupa o cargo há menos de 3 anos, sendo que para os dois grupos, cerca de 25% tem até um ano, e aproximadamente 35% têm entre 2 e 3 anos na função.

Considerando somente os supervisores que cursaram a residência em MFC, 25,6% estão atualmente vinculados a um PRMFC no mesmo município em que nasceram. O mesmo acontece para 43,1% dos preceptores que fizeram residência em MFC e para 33% dos residentes, sendo que a análise indica que essas proporções entre os grupos são significativamente diferentes. Em relação à graduação, para os três grupos, cerca de 50% dos participantes estão vinculados a um PRMFC no mesmo município de sua graduação. Em termos de ter vínculo com um PRMFC em um município diferente do município onde nasceu, cursou a graduação e a residência – no caso de supervisores e preceptores – supervisores (35,6%) e residentes (42,6%) têm os maiores percentuais e a diferença entre as proporções dos três grupos é significativa. Além disso, 41,1% dos supervisores e 73,1% dos preceptores atualmente estão vinculados a um PRMFC no mesmo município onde foram residentes, sendo que a análise indica que as proporções são significativamente diferentes (Tabela 4).

**Tabela 4:** Migração dos supervisores e preceptores que cursaram residência em MFC e médicos residentes. Brasil, 2020.

Município	Supervisor (n = 90)	Preceptor (n = 130)	Residente^a^ (n = 542)	p
Está no mesmo município de nascimento.	23 (25,6)	56 (43,1)	179 (33,0)	0,020
Está no mesmo município da graduação.	40 (44,4)	65 (50,0)	263 (48,5)	0,706
Está no mesmo município onde realizou a residência em MFC.	37 (41,1)	95 (73,1)	-	< 0,001
Está em município diferente dos municípios de nascimento, graduação e realização da residência.	32 (35,6)	23 (17,7)	231 (42,6)^a^	< 0,001

Fonte: Elaboração a partir dos dados da pesquisa “Caracterização dos Programas de Residência em Medicina de Família e Comunidade no Brasil”.

^a^ Para residentes, considera-se município diferente dos municípios de nascimento e graduação.

MFC: Medicina de Família e Comunidade.

A maioria dos residentes está em regiões metropolitanas (n = 327; 60,1%), sendo que, entre eles, pouco mais da metade permanece no mesmo município da graduação (n = 173; 53,1%). Já entre os residentes em que o PRMFC é no interior, a maioria mudou de município entre a graduação e a residência (n = 126; 58,3%).

Quando analisado o tipo de município dos participantes que cursaram residência em MFC, não há diferença significativa entre os grupos (p-valor = 0,702), sendo que 51,1% dos supervisores (n = 47) e 54,6% dos preceptores (n = 71) estão vinculados a PRMFC em regiões metropolitanas. Entre os supervisores que atualmente estão em regiões metropolitanas, aproximadamente metade (n = 22; 48,9%) se fixou no município da residência, enquanto entre os supervisores que estão atualmente em municípios do interior a fixação é menor (n = 15; 33,3%), sendo que dois supervisores não informaram o local em que cursaram a residência. Entre os preceptores, que possuem maior percentual de fixação do que os supervisores, a permanência no mesmo município da residência também é maior quando o município é uma região metropolitana (n = 56; 78,8%) do que entre os preceptores do interior (n = 39; 66,1%).

A regressão de Poisson foi utilizada para avaliar a associação entre a fixação de supervisores e preceptores que cursaram a residência em MFC em relação ao município onde estão atualmente vinculados e às características: grupo; gênero; idade; estado civil; tipo de município onde está atualmente; indicador de mudança entre municípios onde cursou a graduação e a residência. Com o ajuste do modelo, somente as variáveis grupo e migração em relação ao município de graduação se mostraram significativas. Assim, é possível concluir que a fixação de preceptores é 1,7 vezes a de supervisores (IC95% 1,14–2,49), mantendo as demais características constantes. Da mesma forma, a prevalência de fixação para pessoas que mudaram de município entre a graduação e a residência é 1,5 vezes a prevalência entre pessoas que não mudaram de município, com intervalo de confiança entre 1,07 e 2,22, mantendo as demais características constantes. Entre os supervisores e preceptores que cursaram residência em MFC e se fixaram no município (41,1% e 73,1% respectivamente), a maior parte está em capitais ou região metropolitana (59,5% e 58,9%, respectivamente) (Tabela 4).

Por outro lado, no que se refere aos supervisores que mudaram de município (n = 53; 58,9%) entre a residência em MFC e seu local atual, 40,4% cursaram a residência na capital ou região metropolitana e migraram para um município no interior; 7,7% fizeram o caminho inverso; 36,5% estavam em uma capital ou região metropolitana e se mudaram para outro município do mesmo tipo; e 15,4% dos supervisores mudaram entre municípios do interior. O mesmo acontece para os preceptores que mudaram de município (n = 35; 26,9%) entre a residência em MFC e seu local atual: 40% migraram para o interior; 5,7% foram do interior para a capital ou região metropolitana; 37,1% mudaram entre capitais ou região metropolitana; e 17,1% mudaram entre municípios do interior.

Uma parcela dos supervisores que cursou a residência MFC (n = 22; 24,4%) está atuando no mesmo PRMFC onde foi residente, o que representa 59,5% dos que permaneceram no mesmo município em que cursaram a residência. Entre os preceptores, a maioria (n = 78; 60%) está atuando no mesmo PRMFC em que cursou a residência, representando 82,1% dos que permaneceram no município.

A maioria dos supervisores que não são MFC está no interior (n = 21; 77,8%), sendo que apenas 38,1% desses estão no mesmo município em que cursou a graduação. Entre os que estão na capital ou região metropolitana, 50% permanecem no município da graduação. O mesmo ocorre entre os supervisores que são MFC somente por título: a maioria está no interior (n = 22; 62,9%) e 13,6% deles estão no mesmo município em que cursaram a graduação. Entre os que estão na capital, 46,2% permanecem no município da graduação. Tanto entre os preceptores que não são MFC quanto entre os que são especialistas somente por título, cerca de 55% está em regiões metropolitanas. Entre os que não são MFC, aproximadamente 40% permaneceram no município da graduação. Já entre os especialistas por título, 50% dos que estão atualmente em capitais ou região metropolitana permaneceram no mesmo município, e somente 15,4% dos preceptores que estão no interior se fixaram no município da graduação.

## DISCUSSÃO

Caracterizar o perfil e a migração dos integrantes da residência médica pode subsidiar as entidades representativas na formulação e reordenação de políticas adequadas e compatíveis com a realidade, especialmente no que tange à formação de recursos humanos especializados. Isso se refere especialmente ao campo da MFC, considerando que no Brasil existem poucos profissionais com essa formação^10^.

A maioria dos participantes (60,9%) é do sexo feminino, retratando a presença feminina na especialidade MFC, o que também é observado em países como Canadá, Espanha e Reino Unido^11^, assim como em outras áreas da medicina, demonstrando a feminização da carreira médica^3,10,14^. A média de idade dos médicos no Brasil é de 44,6 anos, sendo de 41,7 entre os MFC^3^, o que se aproxima dos dados dos EUA^15^, onde a faixa etária dos MFC é concordante com as médias encontradas nesta pesquisa entre os supervisores e os preceptores.

A pesquisa demonstra mudanças em relação às instituições formadoras de médicos, sendo que a maioria dos supervisores e preceptores cursou sua graduação em uma instituição pública federal ou estadual. Já a maior parte dos residentes (59,1%) cursou em uma instituição privada filantrópica ou comunitária. Esses dados corroboram o estudo que avaliou a distribuição e a expansão dos cursos de medicina no Brasil, observando-se que a maioria das escolas (58,5%) e das vagas de graduação (65,8%) estavam sob gestão privada^16^.

Diante da formação de médicos especialistas, o estudo observou que 17,5% dos participantes supervisores não possuem título em MFC. Desconhece-se na literatura dados comparativos, mas se acredita que esse percentual seja elevado, considerando-se a legislação brasileira que descreve que o supervisor e os preceptores do PRMFC deverão preferencialmente ser especialistas com certificado de residência em MFC e/ou certificação da especialidade acreditado ou expedido pela SBMFC.

Podemos perceber que 10,2% dos preceptores respondentes não possuem a especialidade MFC, e 7,3% não possuem nenhuma especialidade, o que é preocupante, tendo em vista a qualidade da formação do residente^17^. A European Academy of Teachers in General Practice (EURACT), formada por preceptores em MFC, com longa trajetória no ensino em serviço^18^ e preocupados com a qualidade da formação, recomenda que o preceptor tenha treinamento comprovado na especialidade e formação docente concluída, entre outros critérios^19^. Pelo exposto, a especialização e formação docente são pontos fundamentais, que devem ser estimulados e, até mesmo, exigidos na seleção de preceptores dos PRMFC.

Segundo a Demografia Médica no Brasil 2020, o país tem um quantitativo de médicos (2,38 médicos por 1.000 habitantes) próximo aos países desenvolvidos, como os EUA (2,6), Canadá (2,8) e Reino Unido (3)^3,11^. Apesar desse quantitativo, o Brasil ainda apresenta grande desigualdade na distribuição da população médica entre os diversos níveis geográficos e entre as diferentes especialidades médicas^3,20,21^. Quanto à distribuição segundo a especialidade, há um número insuficiente de médicos em especialidades consideradas estratégicas para o SUS, como é o caso da MFC, que em 2020 contava com apenas 7.149 médicos, ou seja, 1,7% do total de especialistas do país, razão de 3,4 para cada 100.000 habitantes^3,22^, ao contrário do que se encontra em outros países, como na Espanha, onde 31% dos médicos estão trabalhando na APS, razão de 87,84 médicos para cada 100.000 habitantes^12^.

Reforçando as desigualdades na distribuição dos médicos pelo território nacional, há capitais com mais de 13 médicos por 1.000 habitantes e regiões do interior do Nordeste com valores inferiores a 1 médico por 1.000 habitantes^3^. Esses dados são reafirmados na pesquisa, em que a vinculação profissional dos participantes revela que mais da metade dos médicos preceptores e residentes está vinculada às capitais do país. Essa é uma tendência mundial de migração de profissionais de saúde em que a fixação tem se concentrado em grandes centros urbanos e polos econômicos, com melhores condições de trabalho, melhor infraestrutura e equipamentos, além de serem locais de concentração de estabelecimentos de ensino e saúde^10,21,23^.

Para preceptores, observa-se uma elevada taxa de fixação no mesmo município em que realizaram o PRMFC (73,1%). Esse resultado, assim como evidenciado na literatura, mostra que o local da residência favorece a fixação dos profissionais médicos, que tendem a permanecer no local onde realizaram sua residência^10,21,24,25^. Entretanto, quando analisado o grupo dos supervisores, a maioria migrou de município após realizar a residência em MFC, sendo que 40,4% migraram de capitais/região metropolitana para o interior, o que pode indicar que a expansão de PRMFC em locais além das capitais pode contribuir na fixação de médicos supervisores, na medida que é necessária a presença de supervisor para o funcionamento do programa.

Para a maioria dos médicos residentes, o local da residência coincide com o local de nascimento e/ou graduação (57,4%), sendo que 48,5% estão no mesmo local da sua graduação. Silva et al.^26^ também identificaram essa tendência, revelando que 37% dos alunos desejam se fixar no mesmo local da graduação, 28% desejam voltar para cidade de origem de nascimento e 20% desejam morar em centro urbano menor. Esses achados reforçam a importância de se considerar a distribuição geográfica das faculdades de medicina e dos programas de residência médica^21,27^.

Quanto ao tempo de atuação, percebe-se que mais da metade dos supervisores e preceptores está atuando na função há menos de 3 anos. Podemos inferir que há uma moderada rotatividade nessas funções, o que pode refletir uma possível falta de valorização e suporte às funções desses profissionais. No caso dos preceptores, desde 2016 há políticas de formação que estimulam a especialização do preceptor, especialmente na MFC, objetivando garantir a oferta de médicos com formação específica em preceptoria, suficiente para a expansão, com qualidade, da residência em MFC^9^. Acredita-se que esse tipo de formação possa fixar o profissional na função de preceptor.

Conclui-se, então, que a pesquisa atingiu seu objetivo de caracterizar o perfil sociodemográfico e analisar as características migratórias dos integrantes dos PRMFC em 2020 no Brasil, apontando temas importantes para o fortalecimento da APS, como o provimento e fixação de especialistas em MFC, que, na sua maioria, são do sexo feminino, adultos jovens e, mais recentemente, formados por instituições de ensino privado, que têm buscado formação complementar para além da residência de MFC e desempenham mais de uma atividade de trabalho.

Quanto à fixação dos participantes, médicos residentes estão vinculados aos grandes centros urbanos, muitos no mesmo local de nascimento e/ou graduação. Para os preceptores, a fixação ocorre principalmente no mesmo município de realização da residência em MFC. Contudo, quando ocorre, o processo de migração do município em que realizou a residência é maior para municípios do interior do que para a capital e região metropolitana.

Uma das limitações deste estudo é o fato de que a proporção de respondentes de algumas regiões foi, em termos relativos, maior em comparação com outras regiões. Também é importante ressaltar que se trata de um estudo transversal, o que torna estático o retrato da análise dos participantes em um contexto que se modifica.

Ainda assim, os dados apresentados no estudo reforçam a necessidade de diálogo permanente e estratégias que envolvam os diferentes níveis de gestão, na tentativa de viabilizar e ampliar políticas de recursos humanos em MFC e de qualificação dos profissionais envolvidos com os programas de residência, como preceptores e supervisores. Por fim, recomenda políticas que priorizem medidas para promover a migração de residentes para PRMFC que estão distribuídos em locais fora das capitais e regiões metropolitanas, bem como estimular a fixação nesses locais, para que possam contribuir com a distribuição e provisão de médicos onde ainda é necessário.
